# Fibre-Reinforced Polymer Reinforced Concrete Members under Elevated Temperatures: A Review on Structural Performance

**DOI:** 10.3390/polym14030472

**Published:** 2022-01-25

**Authors:** Fariborz Sharifianjazi, Parham Zeydi, Milad Bazli, Amirhossein Esmaeilkhanian, Roozbeh Rahmani, Leila Bazli, Samad Khaksar

**Affiliations:** 1 School of Science and Technology, The University of Georgia, Tbilisi 0159, Georgia; f.sharifianjazi@ug.edu.ge (F.S.); s.khaksar@ug.edu.ge (S.K.); 2 Department of Technical and Engineering, Parsian Institute of Higher Education, Qazvin 3471991984, Iran; parhamzeydi@gmail.com; 3 College of Engineering, IT & Environment, Charles Darwin University, Darwin 0801, Australia; 4 School of Mechanical and Mining Engineering, The University of Queensland, Brisbane 4000, Australia; 5 Department of Materials and Metallurgical Engineering, Amirkabir University of Technology, Tehran 1411713136, Iran; esmaeilkhanian-a@aut.ac.ir; 6 Faculty of Civil Engineering, University of Tabriz, Tabriz 5166614711, Iran; roozbeh.rahmani2000@gmail.com; 7 School of Metallurgy and Materials Engineering, Iran University of Science and Technology, Tehran 1411713136, Iran; leilabazli64@gmail.com

**Keywords:** fibre-reinforced polymer, FRP strengthening, FRP-reinforced concrete, elevated temperature, fire

## Abstract

Several experimental and numerical studies have been conducted to address the structural performance of FRP-reinforced/strengthened concrete structures under and after exposure to elevated temperatures. The present paper reviews over 100 research studies focused on the structural responses of different FRP-reinforced/strengthened concrete structures after exposure to elevated temperatures, ranging from ambient temperatures to flame. Different structural systems were considered, including FRP laminate bonded to concrete, FRP-reinforced concrete, FRP-wrapped concrete, and concrete-filled FRP tubes. According to the reported data, it is generally accepted that, in the case of insignificant resin in the post curing process, as the temperature increases, the ultimate strength, bond strength, and structure stiffness reduce, especially when the glass transition temperature *T_g_* of the resin is approached and exceeded. However, in the case of post curing, resin appears to preserve its mechanical properties at high temperatures, which results in the appropriate structural performance of FRP-reinforced/strengthened members at high temperatures that are below the resin decomposition temperature *T_d_*. Given the research gaps, recommendations for future studies have been presented. The discussions, findings, and comparisons presented in this review paper will help designers and researchers to better understand the performance of concrete structures that are reinforced/strengthened with FRPs under elevated temperatures and consider appropriate approaches when designing such structures.

## 1. Introduction

Fibre-reinforced polymer is a composite material that consists of a polymer matrix and fibre reinforcement. Numerous FRPs have been produced, including basalt fibre-reinforced polymers (BFRP), glass fibre-reinforced polymers (GFRP), aramid fibre-reinforced polymers (AFRP), and carbon fibre-reinforced polymers (CFRP) [1,2,3,4]. FRPs have exceptional properties, including light weight, high strength, electrical insulation, low thermal conductivity, impact resistance, dimensional stability, corrosion resistance, and they are non-magnetic [5,6,7,8]. Due to the significant advantages of FRPs over conventional construction materials, including steel and concrete, for retrofitting and strengthening concrete structures, they have gained attention as viable alternatives for reinforcing and retrofitting concrete structures [8,9,10]. These composites are typically employed as “externally bonded” systems to increase the axial sectional, flexural, torsion, and shear capacities of the structural elements of the reinforced concrete, increase the structural members’ stability and serviceability, and provide additional confinement [11,12,13]. Two distinct types of strengthening methods for FRP-reinforced concrete (FRP-RC) are presented: the first makes use of FRP sheets and/or plates and the second uses near-surface mounted (NSM) bars [14]. To prepare the external surface of the concrete for FRP plates and sheets, either high-pressure jet washing or sandblasting is used. Following that, FRP products are used on the concrete surface [15,16,17,18]. This form of external reinforcement is simple and quick to implement. Three types of FRP reinforcements are available for new structures: (1) internal reinforcement with FRP bars; (2) FRP formwork for RC members that stays in place; and (3) FRP tendons for prestressed concrete (PC) components [19,20,21]. 

Due to concerns about the performance of FRPs at high temperatures, the widespread application of FRP-RC in structures has been limited [22,23,24]. In general, prolonged contact with temperatures around and above the glass transition temperature (*T_g_*) of the resin degrades the mechanical properties of FRP materials [25]. *T_g_* is normally represented by a single *T_g_* (often between 50 and 120 °C for resins cured at ambient temperatures), which can be evaluated experimentally using differential scanning calorimetry (DSC) or determined by dynamic mechanical analyses (DMA) [17,26]. In design decision-making, *T_g_* is frequently used as a “critical temperature”, although mechanical performance degrades prior to reaching *T_g_* [27]. When subjected to elevated temperatures (usually greater than 300–400 °C), the thermal decomposition of the FRP organic matrix occurs, potentially emitting smoke, soot, toxic/combustible volatiles, and heat [7,28,29,30]. Organic fibres (e.g., biofibres, PBO, and aramid) employed to strengthen some polymer composites may also degrade and form smoke, fumes, and heat [31,32]. These decomposition processes often result in the further deterioration of the physical and mechanical properties of FRPs due to the degradation of the matrix and, in certain circumstances, the fibres [6,33,34]. Deuring [35] is one of the leading researchers conducting fire tests on externally reinforced concrete beams. According to Deuring’s report, unprotected beams that were strengthened with FRP could withstand a fire for 81 min. In comparison, a similar beam with protected FRP systems could withstand a fire for 146 min. Williams et al. [36] conducted a more recent investigation, in which they tested the performance of CFRP-strengthened RC T-beams under normal fire conditions. The beams were insulated with vermiculite gypsum (VG) insulation. The findings of this experiment revealed that FRP and reinforcing steel components can be kept at a temperature below the critical value necessary to retain their structural integrity by using a suitably insulated system. 

The purpose of this review is to summarise and discuss the findings of investigations on the performance and mechanical properties of FRP-reinforced/strengthened concrete members under elevated temperatures.

## 2. Mechanical Properties of Individual Components at Elevated Temperature

### 2.1. FRP

FRPs exhibit significantly different behaviours from steel or concrete at high temperatures. When exposed to a substantial amount of heat, all polymer matrix composites will burn. Additionally, matrix elements, such as epoxy, vinylester, and polyester, not only facilitate burning but also produce huge amounts of dense black smoke [37]. Furthermore, FRPs degrade in terms of stiffness, strength, and bond characteristics when exposed to even mildly elevated temperatures [38,39]. Numerous research investigations focusing on the mechanical characteristics of FRPs and their constituents at elevated temperatures have been published in the literature [40,41,42,43,44].

**Figure 1:**
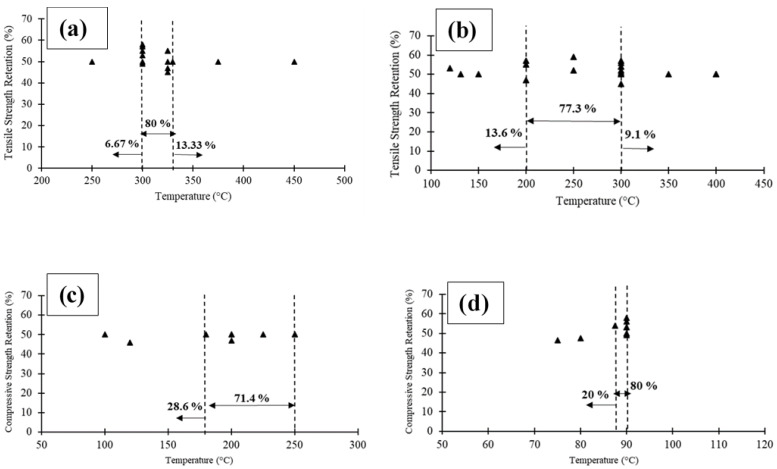
The FRP strength retention versus critical temperature as reported in the literature [50]: (**a**) FRP bars; (**b**) FRP laminates; (**c**) FRP laminates; and (**d**) pultruded FRP profiles.

When elevated temperatures below *T_g_* are applied to the resin matrix, the resin matrix remains relatively unaffected (i.e., some microcracks may form) and the surface of the resin matrix remains rough and similar to that of the unconditioned sample [45]. In this situation, no significant changes occur in the strength or stiffness of the FRP composites. When FRP composites reach their *T_g_*, the resin undergoes a phase transition from glassy to rubbery. In this instance, the FRP materials soften and creep, resulting in a significant loss of stiffness and strength [46]. It has been observed that when FRP materials are subjected to temperatures that are near the resin’s decomposition temperature, their organic matrix decomposes, which emits heat, soot, smoke, and hazardous volatiles. Exposure to such high temperatures (e.g., 300–500 °C) causes the breakage of the modular chains of the resin, chemical bonds, and bonds between the fibres [47,48]. At higher temperatures, the composites ignite and burn. The critical temperature (i.e., the temperature at which 50% of the strength is lost) was reported to be typically between 87–90 °C for pultruded GFRP profiles in compression, 300–330 °C for FRP reinforcing bars in tension, 180–250 °C for laminates in bending, and 200–300 °C for laminates in tension [49]. Figure 1 shows the reported critical temperatures in the literature (i.e., the temperatures that are equivalent to an approximately 50% reduction in mechanical properties) for different FRP composites under different loading conditions.

The compression and interlaminar shear failure of FRP composites occurs at substantially lower loads and temperatures than flexure and tension [50]. Elevated temperatures have a lesser effect on the elastic modulus of FRP composites than on the corresponding strength values. This is mostly due to the fact that the elastic modulus of FRP composites is more closely related to the elastic modulus of the fibres than to the elastic modulus of the resin [49].

A few of the parameters that influence the properties of FRPs are the configuration of fibres and resin, the production technique, and the quality control of the final products. The stiffness and strength properties of FRPs decrease with increasing temperature, although there are considerable variations in the results due to the wide variety of fibre volume fractions, formulations of the matrix, and fibre orientations represented in the data [33,51].

### 2.2. Concrete 

While concrete generally has a high resistance to fire, its mechanical characteristics, such as elastic modulus and strength, degrade when exposed to high temperatures. At elevated temperatures, the failure is mostly due to the creation of cracks that are parallel to the heated surface, changes in the chemistry, and an increase in pore pressure owing to water evaporation. At elevated temperatures, concrete undergoes a variety of physical (vapor diffusion, evaporation, phase expansion, and condensation), chemical (dehydration and thermo-chemical damage), and mechanical (cracking, spalling, and thermo-mechanical damage) phenomena that degrade its qualities [52,53]. The water on the surface of the concrete and capillary water evaporates as the temperature rises and this process is hastened by the reduced cohesive interactions between the water molecules, which is caused by water expansion. At 105 °C, the free water begins to evaporate rapidly. The dehydration of ettringite occurs between 80 and 150 °C, followed by gypsum decomposition between 150 and 170 °C. When the temperature approaches 300 °C, the evaporation of the chemically bound water begins, which reduces the compressive strength of concrete [31,36,54,55,56,57]. Portlandite decomposes between 400 and 540 °C as the temperature increases further. When the temperature of the concrete exceeds 400 °C, the strength of the concrete deteriorates more rapidly due to the breakdown of calcium–silica–hydrate (C–S–H). Between 600 and 800 °C, the second phase of the C–S–H decomposes to create β-dicalcium silicate (β-C_2_S). At 900 °C, the C–S–H fully degrades. As a result, the critical temperature range for concrete is around 400–900 °C and concrete loses the majority of its strength within this range [58]. 

## 3. FRP-RC Structural Members

**Figure 2:**
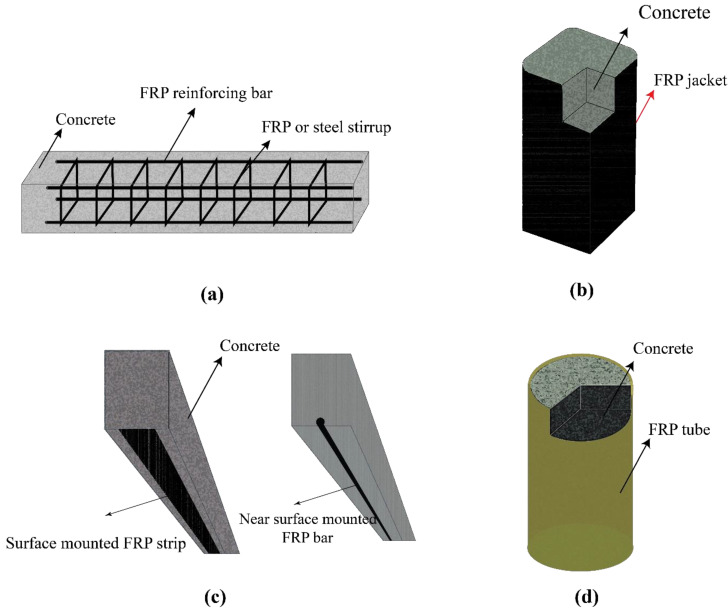
The common applications of concrete members that are reinforced/strengthened with FRP [14]: (**a**) an FRP-reinforced concrete member; (**b**) an FRP-wrapped concrete member; (**c**) an FRP–NSM strip/bar; (**d**) a concrete filled FRP tube. Reproduced from [14], with permission from Elsevier, 2022.

FRPs and concrete could be used to construct different structural systems [59,60,61]. Figure 2 shows the common applications of using FRPs together with concrete elements. However, owing to the unique advantages of such hybrid structures, many other applications could be considered, especially in offshore infrastructures. In this section, the studies that focused on each of the systems shown in Figure 2 are reviewed and discussed in detail.

### 3.1. FRP Laminate Bonded to Concrete

The majority of options for fibre-reinforced strengthening by external bonding are based on polymer systems, most notably those of epoxies [62]. Typically, they serve as the matrix for FRP laminates; nevertheless, they are occasionally used as a primer for the substrate. Their relatively low *T_g_* is one disadvantage of epoxies, which may occur around 40–50 °C for the commercially available epoxy resins that are used in structural engineering. At that temperature, the epoxy polymer transforms from a stiff to a viscous (soft) state, reducing the adhesion between the reinforced element and the FRP laminate. As a result, the majority of EBR–FRP solutions are vulnerable to high temperatures. It is not usually clear whether the issue is limited to fire conditions or whether it may also occur as a result of sun exposure [63,64,65,66].

Thermal loading affects the bond behaviour of the interface of FRP and concrete in two ways, which influences the stress transmission between the FRP and the concrete as well as the load-bearing capacity of the FRP strengthening system. Property changes in the bonding adhesive and the two adhesives induced by the temperature are caused by the first effect, whereas the second effect is caused by the thermal incompatibility of FRP and concrete. The first effect is due to the low *T_g_* of bonding adhesives that are cured at the ambient temperature (commonly epoxy resins), which is normally between 45 and 80 °C. Upon service temperatures above the *T_g_* value, the adhesive changes from a solid to a viscous state, resulting in a loss of strength and stiffness. Such bonding adhesive property degradations impair the bond behaviour of the interface, resulting in the stress transfer loss between materials and the early debonding failure of the FRP laminate [66,67,68]. The second effect (thermal stress effect), on the other hand, is primarily associated with the fact that the concrete and the FRP laminate have different thermal expansion coefficients (CTEs). Due to the determination of the longitudinal CTE of the FRP laminate by the thermal expansion of the fibres, it is different from the longitudinal CTE of concrete. This value for the CFRP laminates that are extensively employed for reinforcing purposes, for example, is near to zero, whereas the CTE of concrete is approximately 10 × 10^−6^/°C at ambient temperatures. Consequently, the aforementioned thermal incompatibility could cause large thermal stresses at the interface of these two materials, thereby affecting the load-bearing capacity of the strengthened structures [69]. 

This issue is not limited to fire conditions; it can also develop after being warmed by the sun’s rays. In a study by Krzywoń [70], concrete beams were strengthened with an externally bonded CFRP strip and the beams were then heated with linear infrared radiators on the strengthened side and loaded to failure using the bending test. Noticeable temperature effects appeared from about 50 °C. As the temperature increased from room temperature to 73 °C, the failure mode changed from concrete debonding to adhesive debonding; the failure moment also decreased from 72.5 to 55.4 (kNm). Failure was followed by delamination in all cases. CFRP strip delamination happened suddenly and unexpectedly, with no obvious warning signs. The significant bearing capacity loss appeared at temperatures over 65 °C. It has been reported that, under service load conditions, the insulated beams and slabs that are strengthened with CFRP can endure four and three hours of standard fire exposure, respectively, thanks to the fire insulation [71].

Despite the vulnerability of epoxy resin to elevated temperatures, post curing of resin during exposure to elevated temperatures may cause some strength improvement [27]. It was reported that the post curing of CFRP-strengthened reinforced concrete beams can change its performance at elevated temperatures. The results demonstrated that when exposed to high temperatures, post curing an FRP system could be an efficient way to improve the performance of the beams that were strengthened with FRPs. The failure of the epoxy layer started at 120 °C and 170 °C for the uncured and post cured FRP systems, respectively. In the sustained load/increasing temperature tests, the failure mode for uncured and post cured samples were different. The post curing of the FRPs appears to preserve the strength of epoxy at high temperatures. FRP debonding and rupture were the failure cause of the uncured members, while the failure of the post cured specimens was due to concrete delamination and no FRP rupture in higher-temperature testing was observed [72].

In order to numerically model FRP reinforced concrete structures, concrete is often simulated as a nonlieanr material using a model derived by Williams and Warnke [73]. In their models, nonlinerity is tacken into acount considering concrte cracking in tension, and crushing in compression, as well as any internal reinforcement plasticity development [74]. These nonlinear effects are often considered by a multi-nonlinear stress-strain curve. The model proposed by Hognestad et al. [75] is one of the most popular concrete stress-strain curve:

(1) $$f_{c}=f_{c}^{\prime }\left[\frac{2\varepsilon_{c}}{\varepsilon_{co}}-{\left(\frac{\varepsilon_{c}}{\varepsilon_{co}}\right)}^{2}\right]\;0\leq \varepsilon_{c}\leq \varepsilon_{co}$$

(2) $$f_{c}=f_{c}^{\prime }-\left(\frac{0.15f_{c}^{\prime }}{\varepsilon_{c}-\varepsilon_{co}}\right)\;\varepsilon_{c}>\varepsilon_{co}$$

where, $f_{c}^{\prime }$ is concrete compressive strength (MPa), $f_{c}$ is concrete compressive stress (MPa) corresponding to the strain value $\varepsilon_{c}$, and $\varepsilon_{co}=\frac{2f_{c}^{\prime }}{E_{c}}$.

In terms of modeling FRP composites, the material characteristics of FRPs are normally considered orthotropic and elastic until reaching to the ultimate strength, which it drops to zero [76]. The bond between FRP and concrete can also be modeled using commonly accepted bond-slip models with proposed by different researchers/standards, such as CEB-FIP model [77]:

(3) $$\tau =\tau_{u}{\left(\frac{S}{S_{u}}\right)}^{0.4}$$

where, *τ* is the bond stress corresponding to the given slip (*S*) in (MPa), $\tau_{u}$ is the maximum bond stress in (MPa), *S* is the relative slip corresponding to a given shear stress in (mm), and $S_{u}$ is the ultimate slip corresponding to $\tau_{u}$ in (mm).

Additionally, a suite of numerical fire simulation software is also being developed in the experimental program, [78,79]. Complex one- and two-dimensional finite difference heat transfer algorithms are combined with strain compatibility–equilibrium evaluations in the numerical models. Models have been created to estimate the load capacity variation and heat transfer behaviour of several types of insulated, uninsulated, FRP-strengthened, and un-strengthened reinforced concrete elements under exposure to pre-defined (standard) fire situations. The models can account for a wide range of variables in their analyses, including the sustained applied load magnitude, size and shape (T-beams or rectangular) of specimens, standard fire type, concrete moisture content, concrete aggregate type, steel reinforcement ratios, bar layouts, FRP type, thickness and width, as well as insulation type, configuration, and thickness. The studies can additionally account for insulation and/or FRP delamination at pre-determined times during a fire. The models are being tested against the findings of the fire tests with the goal of using them to anticipate fire endurance and perform parametric studies that could help engineers to develop fire-resistant FRP strengthening systems. Parallel to the experimental program, modelling efforts are also ongoing. Attempts are being made to better understand and simulate the thermal and mechanical properties of FRP materials at elevated temperatures, with a particular emphasis on the thermally induced degradation of the FRP–concrete bond.

Along with standard numerical approaches, a homogenisation approach [80,81] has been developed to overcome the challenges associated with conventional methods when analysing the thermal behaviour of multi-material component systems. It has been demonstrated that the findings acquired using the homogenisation approach agree well with those obtained using the micromechanics approach.

Hawileh et al. [82] established a three-dimensional finite element model of the CFRP-reinforced T-section RC beams that were evaluated by Williams et al. [36]. The FE model, which was developed using a commercial finite element software, takes into consideration the changes in the mechanical and thermal properties of the constituent materials and conducts separate structural and thermal evaluations. The failure of the strengthening system due to delamination or the degradation of the adhesive was simulated using a simple element-killing procedure when the temperature in the CFRP exceeded 250 °C (for which a strength reduction of 50% was assumed) and the shear stress at the interface of CFRP and concrete exceeded 4.5 MPa. The model’s predictions were consistent with experimental temperatures.

Kodur and Ahmed [83] and Ahmed and Kodur [55] both developed numerical procedures for simulating the mechanical and thermal responses of RC beams that were reinforced with CFRP strips when exposed to fire using the EBR technique. The procedures were carried out using a “macroscopic finite element model” that took into account arbitrary thermal insulation, temperature-dependent material properties, load and constraint conditions, loading schemes and fire scenarios, geometric and material nonlinearity, and appropriate failure criteria. Following a 2D heat transfer analysis of the cross-section, the model entailed the following steps: (i) the calculation of the slip strain at the interface of the CFRP and concrete; and (ii) the generation of moment vs. curvature curves for each time step and beam segment, followed by the computation of internal forces and deflections from beam analysis. Comparing the model to the experimental data that was reported by Blontrock et al. [84] and Ahmed and Kodur [85] validated the model. In general, good agreement was found in terms of the time, temperatures, and deflections required for the strengthening system to delaminate. Additionally, it was demonstrated that reaching *T_g_* in the CFRP does not necessarily result in the failure of CFRP-strengthened RC beams and that more realistic fire limit states should be developed. Several further computational models [86,87,88] for FRP-strengthened concrete members have been established and verified using experimental data.

**Figure 3:**
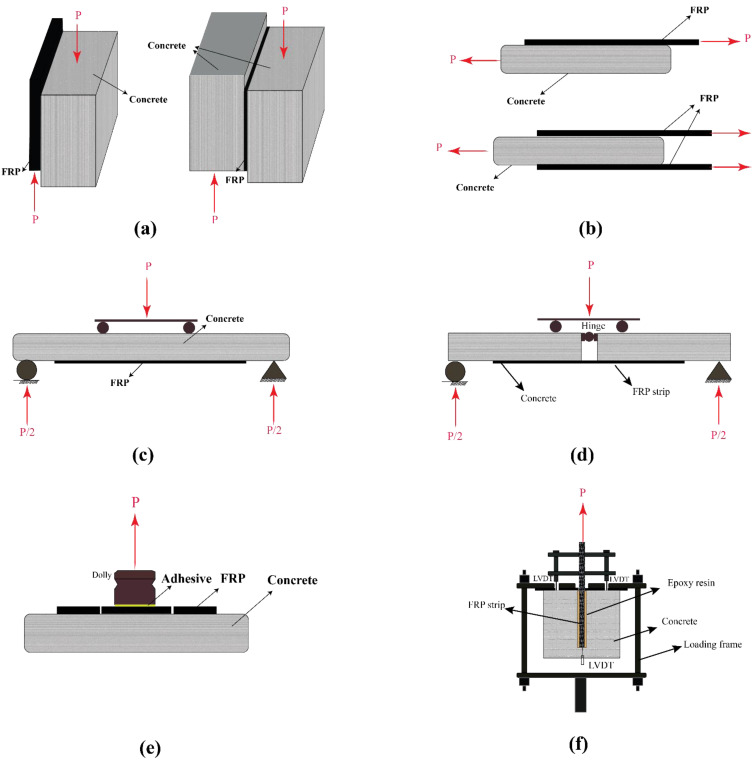
The set-ups that were used to test FRP strips/laminates bonded to concrete: (**a**) shear block; (**b**) lab shear; (**c**) ultimate strength four-point bend; (**d**) bond strength flexural test; (**e**) pull-off; and (**f**) direct pull-out. Reproduced from [14], with permission from Elsevier, 2022.

Table 1 summarises the study plan and results that were reported by several researchers on the structural responses of concrete members that were strengthened with different types of FRP laminates. Different test set-ups were used to study both the ultimate load capacity and the bond strength between the FRP and concrete. Figure 3 schematically shows the different set-ups used by researchers. As seen in Table 1, regardless of the FRP type and laminate thickness, both the bonding strength and the ultimate load-carrying capacity of FRP-strengthened concrete members decrease significantly under moderate and high temperatures, and specifically when the exposure temperature reaches and exceeds the resin glass transition temperature. From Table 1, it can be concluded that the epoxy resin that is used to bond the FRP to concrete plays a key role in the performance of the FRP-bonded concrete systems. Therefore, using resins with appropriate thermo-mechanical characteristics (e.g., high resin glass transition temperature *T_g_* and decomposition temperature *T_d_*) results in a better performance in moderate temperatures (i.e., higher strength retention) and under fire conditions (i.e., longer duration until failure). 

### 3.2. Reinforced Concrete Members

#### 3.2.1. Bond Performance

For transferring loads through the polymer adhesive or matrix, a strong bond between the concrete and the FRP is required [96]. The deterioration of the mechanical characteristics of the matrix material at temperatures exceeding *T_g_* may cause bond loss even at moderately elevated temperatures, which results in the loss of interaction between the FRP and the concrete. In the literature, there have been a lot of studies on the bond characteristics of FRP bars used for concrete strengthening at high temperatures [47,97,98,99]. At temperatures between 100 and 200 °C, the bond strength decreases dramatically to approximately 10% of the room temperature strength because the characteristics of the polymeric matrix at the surface of the rods change. To design concrete members that are reinforced or strengthened with FRP, bond deterioration at elevated temperatures is the main factor to consider. Bond strength reductions in the FRP bars at elevated temperatures are commonly evaluated by bond pull-out tests [23,47]. A noticeable decrement of the bond strength of FRP bars that are embedded in concrete occurs as the temperature at the bond line rises to within the range of the *T_g_* of the bars; considerable bond strength reductions are found at temperatures corresponding to the lowest *T_g_* identified for the bars based on the commencement of a decline in the storage modulus of the bars [97].

The durability of the interface bond between concrete and FRP bars was investigated experimentally, with an emphasis on the deterioration of the surface material of the FRP bar when employing a concrete mix with a high compressive strength. For bond strength tests, 48 pull-out samples were cast, consisting of four distinct types of FRP bars. Additional samples were cast for the concrete compressive strength test. The samples were subjected to the following conditions: tap water kept at 60 °C or room temperature and air that was thermally cycled from −20 to 60 °C. Reductions of 0–20% in bond strength were seen for GFRP bars and reductions of 4–10% were reported for CFRP bars after environmental conditioning. Before reaching the peak bond strength, the conditioning increased the free-end slip. It was reported that the deterioration of the bond primarily resulted from the corrosion of the FRP bar and was less due to the concrete [100].

Several numerical studies were also conducted to evaluate the influence of different parameters and predict the performance of FRP-reinforced concrete [100,101,102,103,104]. Yu and Kodur [105] presented numerical studies that investigated the effect of key parameters on the fire response of FRP rebar-reinforced concrete beams. The research was performed with the help of a macroscopic finite element model that considered the high-temperature properties of constitutive materials, actual load, restraint conditions, and the slip between concrete and FRP rebars that was induced by temperature. According to parametric studies, fire scenario, concrete cover thickness, and rebar type all have a substantial impact on the fire response of FRP rebars-reinforced concrete beams, whereas only a minor impact on the existence of axial restraint is observed. These findings of parametric studies are used to recommend the best insulation schemes to improve the fire resistance of FRP rebar-reinforced concrete beams.

#### 3.2.2. Ultimate Strength

Some research publications have looked into the strength of various FRP-reinforced elements [28,106,107]. Rafi et al. [108] studied behaviour of GFRP and CFRP bar RC beams at high temperatures. The results of fire testing on six simply supported beams made of normal weight concrete were produced. The impacts of different load levels and different FRP bar types were investigated. Over-reinforced beams were constructed and tested in a floor furnace. The failure criterion for the beam was set at 500 °C for the rebar. The temperature distribution over the beam cross-section was found to be nonlinear. The change in temperature in the compression concrete was found to be negligible, and its mechanical properties were almost unaffected. All of the beams passed the failure criterion of a critical rebar temperature of 500 °C. At elevated temperatures, the loss of stiffness in the GFRP and steel RC beams was essentially identical and was unaffected by bar modulus or load levels. When compared to other beams, the CFRP bar-reinforced beams had better stiffness characteristics.

Another study found that shear failure is the most common mode of failure in hybrid concrete beam specimens that are reinforced with GFRP when exposed to temperatures ranging from 300 to 700 °C and subjected to monolithically raised static loads until failure. Around a 53% reduction in the ultimate load capacity was observed in the hybrid reinforced concrete beam when subjected to 700 °C in comparison to ambient temperatures, according to the findings [109]. The fatigue performance of GFRP/CFRP bar-reinforced concrete beams after being subjected to elevated temperatures was also evaluated. To assess the fatigue behaviour of beams, the effects of fatigue load level, high temperature, holding time, and FRP bar type were explored. Below 400 °C, the fatigue life of concrete beams that were reinforced with GFRP was reduced more severely than that of concrete beams that were reinforced with CFRP, and at 600 °C, the bearing capacities of both CFRP- and GFRP-reinforced beams were lost. With the number of fatigue cycles, the development of concrete strain and fracture width, as well as the deflection of the concrete beams that were reinforced with FRP, was accelerated by the raised temperature [110].

Hajiloo et al. [52] performed fire tests on a full-scale concrete slab that was reinforced with GFRP. The loads were equally distributed, which created a flexural moment of 45 kNm. This value was 55% of the slabs’ ultimate moment resistance at ambient temperatures and this flexural moment was maintained throughout the fire test. Both of the loaded slabs were exposed to the ASTM-E119 standard fire for more than three hours. In order to anticipate the deflection behaviour of the FRP-reinforced concrete structures within the range of realistic increased temperatures, Faruqi et al. [111] created a model that incorporates the progressive changes in the elastic modulus of FRP by temperature. The predictions provided by the model were in good agreement with experimental results that were published in the literature. This novel method adds to the tools for assessing the deflection of FRP-reinforced concrete structures in the event of a fire.

Rafi et al. [103] developed a three-dimensional nonlinear finite element model to predict the response of concrete beams that were reinforced with FRP in an elevated temperature regime. The analytical model simulated the propagation of temperature, concrete stresses in beams, and stiffness and strength characteristics. To determine the temperature distribution across the beams, a transient heat transfer analysis was performed. The smeared cracking approach was used for modelling the crack formation and propagation. The models agreed well with the measured temperature, stiffness data, and beam strength; however, its estimation of temperature in sites with a thick concrete layer was conservative.

Lin and Zhang [102] developed a facile two-node layered composite beam element for the accurate simulation of the structural behaviour of steel/FRP-reinforced concrete beams that were subjected to combined thermal and mechanical loading during a fire. The temperature distribution over the cross-section of the beam was determined using a nonlinear finite element analysis based on heat transfer theory. The model was validated using the results presented in [112], and the effects of various parameters on the flexural response of the concrete beams that were reinforced with FRP in fire conditions were also investigated using the current finite element model. These parameters included the level of load, thickness of the concrete cover, and type of FRP reinforcement.

**Figure 4:**
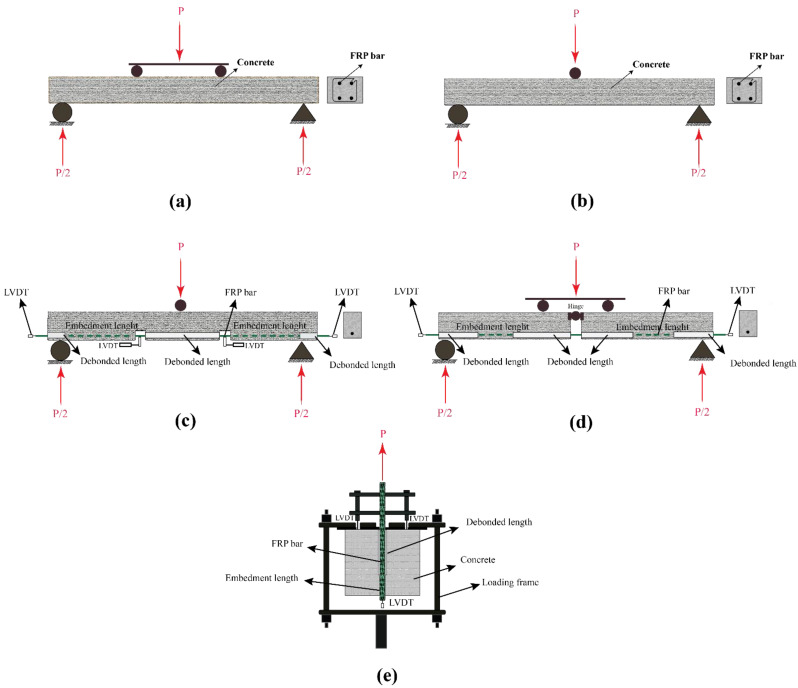
The set-ups that were used to test FRP-reinforced concrete: (**a**) ultimate strength four-point bend; (**b**) ultimate strength three-point bend; (**c**) bond strength three-point bend; (**d**) bond strength four-point bend; and (**e**) direct pull-out. Reproduced from [14], with permission from Elsevier, 2022.

Table 2 summarises the study plan and results that were reported by several researchers on the structural responses of FRP-reinforced concrete members with different types of FRP bars. Different test set-ups were used to study both the ultimate load capacity of the FRP-reinforced concrete members and the bond strength between the FRP bar and the concrete. Figure 4 schematically shows the different set-ups that were used by the researchers. As seen in Table 2 and similar to the results observed in FRP-strengthened concrete structures, the bond strength and the load-carrying capacity of FRP-reinforced concrete members decreases dramatically when the temperature reaches and passes the resin’s *T_g_*. However, it was observed in several studies that under very high temperatures, as well as fire conditions, the structure can still carry a considerable load despite the significant bond strength reduction. This may be due to the fact that FRP bars are embedded in concrete and are not directly exposed to heat and oxygen. 

### 3.3. FRP-Wrapped Concrete Members

Over the past two decades, research projects all over the world have studied the behaviour of externally bonded FRPs that were used for reinforcing RC structures. The exterior of the RC structures is connected to FRPs, often with an epoxy resin adhesive, which provides extra tensile or confining reinforcement, in addition to the internal reinforcing steel. There has now been enough study and execution to produce numerous guidelines and design codes for using FRPs in conjunction with concrete structures [117,118]. In several studies, it has been demonstrated that external FRP wraps on RC columns could greatly improve the strength, as well as the ductility, of these elements [119,120]. As a result, FRPs have been widely used for repairing and restoring RC columns in existing bridges. However, the use of FRP wraps in structures has been limited by concerns about their performance in the case of fire. The majority of FRPs are sensitive to the combustion of the polymer matrix, which can result in the enhanced spread of flames and harmful smoke emissions. Furthermore, at their *T_g_*, the rapid loss of stiffness and strength occurs in commonly used adhesives and polymer matrices. For externally bonded systems, the crucial *T_g_* depends on the individual polymer matrix composition, among other considerations. FRPs may ignite and facilitate the propagation of flames and the production of poisonous smoke if left unprotected in a fire [8], and also rapidly lose bond and/or mechanical performance. This could raise questions about the fire resistance of FRP-strengthened RC columns in buildings where fire is a major design consideration [121].

As a result, much more research is needed to fully comprehend the performance of structures that are strengthened with FRPs in terms of fire resistance. Al-Salloum et al. [121] investigated the behaviour of concrete that was externally confined with CFRP and GFRP sheets using uniaxial compression at high temperatures (exposure time of 1, 2 or 3 h at 100 °C and 200 °C). The results showed that, at slightly above the *T_g_* of the epoxy resin (100 °C), a small loss in strength occurred in both GFRP- and CFRP-wrapped specimens, which resulted from the epoxy melting. At 200 °C, the strength loss was more noticeable. The efficiency of the FRP confining system that was bonded with epoxy diminished significantly (under a steady-state thermal regime and concentric axial compression), but did not vanish as the temperature increased, particularly in the region of the epoxy resin/adhesive *T_g_* [122].

A numerical model for the prediction of the response of FRP-wrapped RC circular columns with thermal insulation to fire was developed by Bisby et al. [31]. The model took into account the temperature-dependent fluctuation of thermo-physical characteristics and consisted of two stages: first, an analysis of finite difference heat transfer; second, an analysis of strain–equilibrium axial load capacity, which was calculated during standard fire exposure. They made the assumption of axisymmetric heat transport and ignored the heating effect of steel rebars. Buckling and crushing strengths were calculated by accounting for the temperature-dependent stress–strain compressive response of concrete along with the FRP wrap-induced confining pressure, using a modified version of Spoelstra and Monti’s confinement model [123]. The output contained load vs. mid-height deflection charts for a variety of fire exposure durations, indicating the axial capacity vs. time relationship. Experimental data reported by Bisby et al. [124] were utilised to validate the model, which was demonstrated to be capable of predicting the axial deflection and temperature profiles of the columns after fire exposure. Chowdhury et al. [125] further validated this model using data from furnace tests on insulated RC columns that were wrapped with FRP. There was a good match between the expected and measured temperatures. Chowdhury et al. [126] modified the previous model to include the structural behaviour of short or thin, eccentrically or concentrically loaded FRP-wrapped RC rectangular columns in both ambient and fire environments. The heat transfer analysis was performed using a finite difference approach that was similar to that employed by Bisby et al. [31].

**Figure 5:**
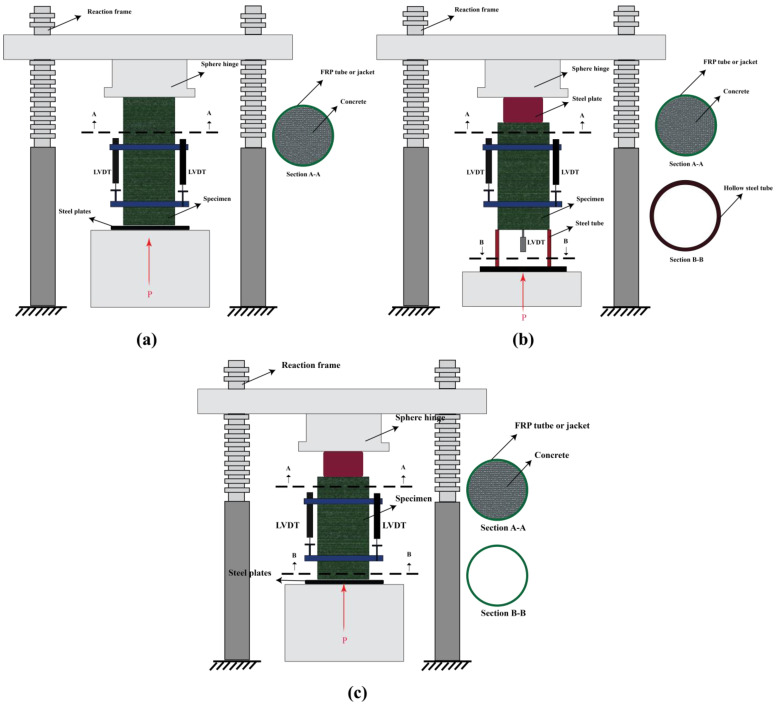
The set-ups that were used to test FRP-wrapped concrete columns and concrete filled FRP tubes: (**a**) compressive; (**b**) push-out test set-up type 1; and (**c**) push-out test set-up type 2. Reproduced from [14], with permission from Elsevier, 2022.

Table 3 summarises the study plan and results that were reported by several researchers on the structural responses of FRP-wrapped concrete members with different types of FRP sheets. The compressive load-carrying capacity and the efficiency of the FRP wrapping were studied by different researchers. Figure 5 shows the test set-ups that were used by the different researchers to study the compressive strength and bond strength of FRP-wrapped concrete columns and concrete filled FRP tubes. From the reported results, it can be concluded that the efficiency of FRP wrapping on the compressive load-carrying capacity of concrete columns under elevated temperatures depends on the fibre type and the number of applied layers. In terms of the FRP layers, it was reported in [122] that, under elevated temperatures, the efficiency of three layers of CFRP wrapping was higher than one layer. In terms of the fibre type, although it was shown in [127] that CFRP-wrapped concrete columns had a better compressive performance compared to the GFRP-wrapped columns, more studies are required to clearly understand the effect of different fibres, particularly GFRP and BFRP. It is also worth mentioning that the effect of fibre orientation could be considered as a potential factor in studying the compressive performance of FRP-wrapped concrete columns under elevated temperatures. 

### 3.4. Concrete Filled FRP Tubes

It is well-known that, in harsh environments, carbon steel tubes are highly susceptible to being corroded after prolonged contact with acid rain, seawater, and other aggressive agents [38,133]. To address such concerns, corrosion-resistant FRP tubes filled with concrete have recently been employed [134,135,136]. Although there are numerous reports addressing the performance of reinforced concrete with FRP bars and externally bounded laminates, as well as FRP-wrapped concrete elements, at elevated temperatures, very few studies have investigated the behaviour of concrete filled FRP tubes at high temperatures. Guo et al. [137] investigated the mechanical characteristics of short, pultruded concrete filled GFRP tubular (CFGT) columns under axial compressive loading at extreme temperatures. Axial testing was performed on short CFGT columns that were subjected to various high temperatures and temperature durations. The concrete compressive strength, GFRP tube thickness, elevated temperature, and temperature duration were the primary variables that were investigated in this work. The findings of the experiments showed that when the temperature rose, the initial stiffness and ultimate strength of the samples decreased significantly whereas ductility increased. When the temperature approached 200 °C, the ultimate strengths of the samples were significantly reduced (approximately 37%). The impact of elevated temperatures on the specimens’ ultimate strength was the most significant among the four main factors, whereas the concrete compressive strength and GFRP tube thickness had some positive impacts, and the effect of temperature duration was negligible. According to the findings of the experiments, a parameter formula for calculating the ultimate strength of the short columns at elevated temperatures was produced, which demonstrated high rationality and accuracy when compared to the test results.

In a similar study conducted by Tabatabaeian et al. [138], the properties of concrete filled pultruded GFRP tubular columns were also assessed at high temperatures. The goal of this study was to see how exposure temperature (25–400 °C), exposure time (60 and 120 min), and the strength of the concrete core (30 and 60 MPa) affected the bond and compressive behaviour of CFGTs. Split disk and compressive tests were used to investigate the performance of unexposed and exposed concrete cores, pultruded GFRP hollow tubes, and CFGTs. To determine the bond-slip strength of the partially filled specimens, the push-out test was also used. The maximum load-bearing capability of stub columns exposed to elevated temperatures was 8%, 22%, 34%, and 51% lower than the unexposed counterparts at temperatures of 100, 200, 300, and 400 °C, respectively. The GFRP tubes withstood around one quarter of the overall load-bearing capability, regardless of the core strength or exposure conditions. Furthermore, it was discovered that when the exposure temperature and concrete core strength increased, the final axial strain (related to tube rupture) of the column samples decreased; however, the ultimate axial strain was not affected by exposure time. To develop novel models for predicting experimental outcomes, the dilation and load-bearing capability of CFGTs were studied. In terms of bond strength testing, it was determined that increasing the exposure temperature increased interlocking and thus, the coefficient of kinetic friction, which resulted in increased bond strength. Eventually, equations for estimating the bond strength of CFGTs after being exposed to high temperatures were presented.

## 4. Summary

A systematic overview and discussion regarding the structural performance of FRP-reinforced/strengthened concrete members after exposure to elevated temperatures was presented. Although FRP-reinforced/strengthened concrete members provide many benefits, their vulnerability to elevated temperatures remains a challenging concern. By reviewing the research conducted on concrete strengthened with FRP composites, one can conclude that their performance when subjected to elevated temperatures is well studied. In addition to the experimental data, analytical models have been developed to predict tensile and bond strength reductions of FRPs at elevated temperatures. In terms of the bond between FRPs and concrete, it has been shown that the thermal loading affects the bond behaviour through influencing (i) the stress transmission between the FRP and the concrete and (ii) the load-bearing capacity provided by the FRP system. It is generally observed that the degradation of the resin’s mechanical characteristics at temperatures exceeding *T_g_* may result in bond loss, even at moderately elevated temperatures (e.g., 90% bond strength reduction at temperatures between 100 and 200 °C), which results in the loss of FRP–concrete interaction. Significant ultimate strength reductions also occur when FRP-reinforced beams are exposed to fire (e.g., 53% flexural strength reduction of GFRP reinforced concrete beam exposed to 700 °C). However, the retention is significantly affected by the concrete cover, FRP bar type and diameter, and the thermal properties of the components. Given the discussion and findings presented in this review, more experimental and numerical studies are needed to develop comprehensive predictive models that are capable of predicting the structural performance of FRP-reinforced/strengthened concrete when exposed to elevated temperatures. The present paper provided a fundamental insight that could be used to develop or enhance new and existing design codes/standards for FRP-reinforced/strengthened structures.

## 5. Recommendations for Future Studies

Sufficient implementations and investigations have now been conducted to develop numerous guidelines and design codes for the application of FRPs in conjunction with concrete structures. Following the discussion presented in this paper, it can be concluded that more experimental and numerical research studies are required to address several research gaps regarding the performance of FRP-reinforced/strengthened concrete members under elevated temperatures. The following research topics are therefore recommended for future studies to fill some of these gaps:

(1) Applying cyclic and impact loading to FRP-reinforced/strengthened concrete members under elevated temperatures in order to study their dynamic behaviour after exposure to elevated temperatures. Currently, most studies have been conducted under static loading.
(2) The current experimental data can be used to verify/calibrate finite element numerical models and then comprehensive parametric studies can be conducted to investigate the effects of different parameters, such as material thermal and mechanical characteristics, resin curing ratio, fibre type and orientation, heating rate, etc.
(3) Conducting tests using real fire. Currently, most studies have been conducted under electrical furnace conditions. It is expected that the performance of structural members under real fire conditions may be significantly different from that of simulated standard fire testing.
(4) Studies on concrete filled FRP tubes under elevated temperatures are very limited. Therefore, several effective parameters, such as fibre type and orientation, tube geometry (e.g., dimeter to thickness ratio), surface friction coefficient (in the case of studying the bond between the concrete and the tube), etc. are yet to be investigated.
(5) Conducting full-scale tests to investigate the effect of stress redistribution and structure size effect.

## Figures and Tables

**Table 1:** The research plan and results summary of the reported structural responses of concrete members strengthened with FRP laminates.

Ref	Study Type	Sample	FRP Type	*T_g_* (°C) of Resin	Exposure Condition	Test Type	Results
[70]	Experimental	Externally bonded concrete beams	A laminate of a single layer CFRP sheet	NA	20–80 °C	Bending test	Significant degradation occurred in the bearing capacity above 65 °C. Failure moment decreased from 72.5 to 55.4 kNm.
[71]	Experimental and numerical	RC flexural members, RC slabs	One layer of CFRP + isolation layer	NA	Fire	Bending test	For four hours, RC beams reinforced with CFRP and supplemented with spray (thickness of 19 and 32 mm) could withstand service load levels. Three hours could be withstood by CFRP-reinforced RC slabs accompanied with fire insulation (thickness of 19 and 25 mm). At temperatures that were far higher than the polymer’s *T_g_*, the complete loss of the CFRP–concrete composite action occurred. The numerical model presented in this research can be used to accurately assess the fire response of the flexural components of CFRP-strengthened concrete.
[89]	Experimental	Concrete prisms	CFRP strips	NA	1 and 2 h at 200, 400, and 600 °C	Single-lap shear	For thermal exposure of 1 h at 200, 400, and 600 °C, the residual bond strength employing epoxy adhesive was 94, 79, and 49%, respectively. For 2 h of exposure, the equivalent values were 86, 75, and 41%, respectively. For temperature exposures of 1 h at 200, 400, and 600 °C, the residual bond strength following the repair of the heat-damaged concrete with CFRP using a cement-based adhesive was 91, 79, and 70%, respectively.
[90]	Experimental	RC prisms	CFRP	68 °C	1 h at 20–150 °C	Double-lap direct shear	At 150 °C, the specimens retained about 17% of their ambient bond strength.
[91]	Experimental and numerical	Concrete blocks	CFRP	NA	Fire	Single shear	The model demonstrated that the epoxy reached the failure point in a relatively short period of time when exposed to normal fire. Additionally, the model was utilised to forecast the required insulation thickness for two- and three-hour fire resistance levels. Experimental data were used to validate the model’s predictions.
[92]	Experimental	Ceiling of a concrete structure	CFRP	60 °C	Fire	Fire	The experiments showed the vulnerability of FRP reinforcement in the event of a compartment fire. The *T_g_* was promptly exceeded in the bonding adhesive in all specimens.
[93]	Numerical	NA	CFRP	NA	20–100 °C	A nonlinear local bond-slip model (double-lap shear)	The interfacial fracture energy (Gf) was nearly constant at first, then began to decline as the temperature approached the *T_g_* of the bonding adhesive. Moreover, the interfacial brittleness index (B) followed a similar pattern.
[94]	Numerical	NA	CFRP	NA	20–90 °C	Single-lap pull-out bond	The normalised value of the interfacial bond characteristic at high temperatures was discovered to be a function of DT (service temperature subtract *T_g_*).
[56]	Experimental	Rectangular concrete specimens	CFRP sheet and laminate and GFRP sheet	55 °C	20–80 °C	Double-face pure shear	With service temperatures exceeding the *T_g_* of the adhesive, the maximum bond stress was reduced. τ_max_ was reduced by 25% in the case of CFRP laminate, 72% in the case of GFRP sheet, and 54% in the case of CFRP sheet at 80 °C compared to room temperature.
[95]	Experimental	Rectangular Recycled Aggregate (RA) concrete	CFRP	NA	23, 400, and 600 °C	Pull-out	The bond load was reduced and slippage was increased when exposed to high temperatures. Concrete separation was the failure mode in all examples.

**Table 2:** The research plan and results summary of the reported structural responses of FRP-reinforced concrete members with FRP bars.

Ref	Study Type	Sample	FRP Type	*T_g_* (°C)	Exposure Condition	Test Type	Results
[99]	Experimental and numerical	Sand-coated GFRP rebars embedded in concrete cylinders	GFRP rebars	98 °C	Tensile: 20–300 °C; and pull-out test: 20–140 °C	Tensile and pull-out tests (steady-state conditions)	With the increasing temperature, the strength and stiffness of the interface of the GFRP concrete were dramatically reduced, especially when the *T_g_* of the GFRP rebars was approached and exceeded.
[98]	Experimental and numerical	A GFRP bar embedded in the center of a cylindrical concrete block	GFRP bars	165 °C	20–300 °C	Pull-out test	The retained bond strength decreased from 100% to 7.2% from 20 °C to 300 °C; the slip at average bond strength decreased from 0.69 mm to 0.24 mm.
[113]	Experimental	A GFRP bar embedded in a cylindrical concrete block	GFRP bars	165 °C	20–300 °C	Pull-out test	For specimens subjected to temperatures near to *T_g_*, the bond strength retention could be as low as 30%, and at 300 °C, it decreased to less than 10%.
[108]	Experimental	An FRP bar embedded in a rectangular concrete block	CFRP and GFRP bars	NA	20–500 °C	Four-point bend test	At elevated temperatures, the stiffness loss in the GFRP and steel RC beams was essentially identical and was unaffected by bar modulus or load levels. When compared to other beams, the CFRP bar-reinforced beams had better stiffness characteristics.
[109]	Experimental and numerical	GFRP-reinforced rectangular concrete beams	GFRP bars	NA	300–700 °C	Three-point bend test	Compared to the ultimate load capacity of the beam at room temperature, that of a GFRP-reinforced concrete beam was reduced by around 53% at 700 °C. Finite element software ABAQUS was utilised to study the effect of some important parameters.
[110]	Experimental and numerical	FRP-reinforced rectangular concrete beams	CFRP and GFRP bars	GFRP: 155 °C CFRP: 139 °C	200–600 °C	Fatigue test (four-point bending)	The fatigue strength of the beams was reduced from 0.12 ultimate load to 0.10 ultimate load after being exposed to 400 °C for 2 h. With a coefficient of variation of 2.8–7.0%, the CEB-FIP model had the best accuracy.
[52]	Experimental	A full-scale FRP-reinforced concrete slab	GFRP bars	113, 118 °C	Fire test for 3 h	Bending test	Under flexural pressure, the reinforced slabs had a fire endurance of almost 3 h. At temperatures around the *T_g_* of the bars, the majority of the bond strength was lost. Despite the fact that the adhesive in the reinforcing bars was entirely burnt, none of the reinforcing bars ruptured.
[114]	Experimental and numerical	A GFRP rebar embedded in cylindrical concrete	GFRP rebars	104, 157 °C	25–300 °C	Steady-state tensile and pull-out tests	The ribbed rebars showed bond strength losses ranging from 20% to 34%, while the sand-coated rebars had a reduction of 81%; at temperatures above the rebars’ *T_g_*, the majority of the GFRP–concrete interaction in the ribbed rebars was reduced.
[115]	Experimental	A GFRP rebar embedded in rectangular concrete	GFRP and CFRP rebars with sand coating treatment	120 °C	Fire, up to 1000 °C for 2 h	Four-point bend test	The concrete beam that were reinforced with carbon and glass rebars of diameters 10 mm and 14 mm reached 66%, 31%, and 46% of their initial load-bearing capacities, respectively.
[116]	Experimental	FRP-reinforced concrete beams	BFRP, hybrid FRP with basalt and carbon fibres (HFRP), and nano-hybrid FRP (nHFRP)	NA	Fire	Post fire: four-point bend test	After being exposed to fire, a reduction in the overall strength capacity of the FRP-reinforced beams was observed by approximately 43%, 40%, and 43% for the beams with tensile zones that were reinforced with BFRP bars, HFRP bars, and nHFRP bars, respectively.

**Table 3:** The research plan and results summary of the reported structural responses of FRP-wrapped concrete members with FRP sheets.

Ref	Study Type	Sample	FRP Type	*T_g_* (°C)	Exposure Condition	Test Type	Results
[122]	Experimental	FRP-wrapped cylindrical concrete (hoop direction)	CFRP sheet (1 and 3 layers)	58 °C	20–400 °C (a steady-state thermal regime)	Concentric axial compression	At ambient temperatures, the strength effectiveness (*f_cc_*/*f_co_*) of a single layer of FRP was approximately 2.02. As the temperature increased, the efficiency of the confinement was reduced. At 150 °C, the single FRP layer’s efficacy was at its lowest (1.13). The *f_cc_*/*f_co_* values for FRP jacketing were 3.89 in the case of three layers at ambient temperature. At 400 °C, the minimum effectiveness for three FRP layers was 2.39.
[128]	Experimental and numerical	FRP-confined square concrete prisms (hoop direction)	BFRP sheet (2, 3, and 4 layers)	NA	200–800 °C	Axial compression test	The tensile rupture of the BFRP jackets was the cause of the failure. The use of BFRP jackets was shown to improve the ultimate axial strain and compressive strength of heat-damaged concrete. The concrete core coated in additional BFRP jacket layers had a greater increase in deformation and strength.
[129]	Experimental	FRP-wrapped circular columns	CFRP sheet (1 layer)	NA	20–800 °C for 3 h	Uniaxial compression test	From room temperature to 800 °C, concrete compressive strength was reduced from 58 to 30.7 MPa.
[127]	Experimental and numerical	FRP-wrapped circular columns	CFRP and GFRP sheets (1 layer)	NA	20–300 °C for 1–3 h	Uniaxial compression test	The wrapped CFRP and GFRP specimens lost about 25.3% and 37.9% of their compressive strength after 3 h of exposure to 300 °C, respectively.
[126]	Experimental and numerical	FRP-wrapped rectangular columns	CFRP sheet (1 layer)	NA	Fire	Uniaxial compression test	Under ambient and fire conditions, a novel computer model was developed to predict several aspects of the structural and thermal response of uninsulated or insulated, slender or short, FRP-wrapped or unwrapped, and eccentrically or concentrically loaded reinforced concrete columns.
[130]	Numerical (artificial neural networks)	FRP-confined concrete column	NA	NA	Fire	ANSYS software	With an overall accuracy of 85–90%, the suggested ANN model could predict FRP, concrete, and steel reinforcement and the temperature during fire exposure.
[131]	Experimental and numerical	FRP-wrapped circular and square columns + insulation layer	CFRP sheet (1 layer)	85 °C	Fire	Full-scale fire resistance test + FORTRAN	Both columns had fire resistance ratings of more than 4 h. The validation of the numerical models created, particularly for circular and square columns, was carried out using experimental results.
[132]	Experimental	Insulated FRP-wrapped square RC columns	CFRP sheet (1 layer)	NA	Fire	Full-scale fire resistance experiments	Fire endurance of 4 h or more was achieved with FRP-strengthened square RC columns protected with the fire protection system mentioned here.

## Data Availability

The data presented in this study are available on request from the corresponding author.

## Abbreviations

FRP	fibre-reinforced polymer
GFRP	glass fibre-reinforced polymer
BFRP	basalt fibre-reinforced polymer
CFRP	carbon fibre-reinforced polymer
HFRP	hybrid fibre-reinforced polymer
NSM	near-surface mounted
PC	prestressed concrete
DSC	differential scanning calorimetry
DMA	dynamic mechanical analyses
*T_g_*	glass transition temperature
*T_d_*	decomposition temperature
PBO	polybenzoxazole
RC	reinforced concrete
VG	vermiculite gypsum
C–S–H	calcium–silica–hydrate
C_2_S	dicalcium silicate
CTE	thermal expansion coefficient
RA	rectangular recycled aggregate
*f_cc_*	peak stress sustained by the confined concrete cylinder
*f_co_*	concrete only strength of the control cylinder
CFGT	concrete filled GFRP tubular
EBR	externally bonded reinforcement
